# High glucose concentration induces endothelial cell proliferation by regulating cyclin‐D2‐related miR‐98

**DOI:** 10.1111/jcmm.12765

**Published:** 2016-02-03

**Authors:** Xin‐Xin Li, Yue‐Mei Liu, You‐Jie Li, Ning Xie, Yun‐Fei Yan, Yong‐Liang Chi, Ling Zhou, Shu‐Yang Xie, Ping‐Yu Wang

**Affiliations:** ^1^ Department of Biochemistry and Molecular BiologyBinzhou Medical UniversityYantaiShandongChina; ^2^ Yantaishan HospitalYantaiShandongChina; ^3^ Shandong China Traditional Medical Affiliated HospitalJinanChina; ^4^ Institute of EpidemiologyBinzhou Medical UniversityYantaiShandongChina

**Keywords:** cyclin D2, miR‐98, cell proliferation, type 2 diabetes, gene expression

## Abstract

Cyclin D2 is involved in the pathology of vascular complications of type 2 diabetes mellitus (T2DM). This study investigated the role of cyclin‐D2‐regulated miRNAs in endothelial cell proliferation of T2DM. Results showed that higher glucose concentration (4.5 g/l) significantly promoted the proliferation of rat aortic endothelial cells (RAOECs), and significantly increased the expression of cyclin D2 and phosphorylation of retinoblastoma 1 (p‐RB1) in RAOECs compared with those under low glucose concentration. The cyclin D2‐3′ untranslated region is targeted by miR‐98, as demonstrated by miRNA analysis software. Western blot also confirmed that cyclin D2 and p‐RB1 expression was regulated by miR‐98. The results indicated that miR‐98 treatment can induce RAOEC apoptosis. The suppression of RAOEC growth by miR‐98 might be related to regulation of Bcl‐2, Bax and Caspase 9 expression. Furthermore, the expression levels of miR‐98 decreased in 4.5 g/l glucose‐treated cells compared with those treated by low glucose concentration. Similarly, the expression of miR‐98 significantly decreased in aortas of established streptozotocin (STZ)‐induced diabetic rat model compared with that in control rats; but cyclin D2 and p‐RB1 levels remarkably increased in aortas of STZ‐induced diabetic rats compared with those in healthy control rats. In conclusion, this study demonstrated that high glucose concentration induces cyclin D2 up‐regulation and miR‐98 down‐regulation in the RAOECs. By regulating cyclin D2, miR‐98 can inhibit human endothelial cell growth, thereby providing novel therapeutic targets for vascular complication of T2DM.

## Introduction

Type 2 diabetes mellitus (T2DM) is a serious chronic metabolic disorder, resulting from the absolute or relative insufficiency of insulin 1, 2. High levels of insulin and glucose in the blood can damage blood vessel function; thus, T2DM is a risk factor for cardiovascular disease. The increased glucose intake by endothelial cells may cause endothelial dysfunction in diabetes 3, 4. T2DM is a source of disability and morbidity owing to vascular complications 5. Approximately, 60–80% of T2DM patients present hypertension, and the vascular complications of T2DM patients account for approximately 60% of all T2DM‐related deaths 6, 7.

The mechanisms underlying the pathology of vascular complications involve inflammatory mediators 8, 9, of which endothelial cells are major regulators. These cells perceive mechanical signals and further convert them into biological events affecting cell proliferation and apoptosis 10, 11. VEGF and its receptors activate pathways leading to endothelial cell proliferation and eventually capillary tube formation 12. In response to growth factors, cyclin D proteins are up‐regulated in proliferating cells 13. Cyclin‐dependent kinase (CDK) complexes can control the G1 to S transition 14, 15, and phosphorylate numerous proteins, resulting in hyperphosphorylation of RB1, thereby promoting cell proliferation 15. Protocatechuic aldehyde treatment reduced cyclin D2 expression in vascular smooth muscle cells, which further induced platelet‐derived growth factor‐stimulated cell proliferation 16, indicating that cyclin D2 is involved in the pathology of vascular complications.

Emerging evidence suggests that microRNAs (miRNAs) participate in the development of diabetes and its vascular complications, and specific miRNAs can modulate epigenetic signatures by targeting methyltransferases 17. In different cell models, increased levels of DNA methyltransferase 1 may result from the activation of hyperglycaemia or reactive oxygen species, which can further influence the role of miR‐125b 18. Elevated miR‐125b levels can suppress methyltransferase Suv39h1 and H3K9me3 expression in the promoter regions of inflammatory genes (MCP‐1 and IL‐6), thereby increasing the levels of these cytokines in myocardial vascular smooth muscle cells of diabetic mice 17. The most feasibly characterized inflammatory miRNA is miR‐146a, which is involved in restraining inflammation and switching off acute inflammation after removal of harmful stimuli 19. The expression of miR‐146 has been associated with several diseases, including diabetes 20, 21, 22. These studies noted that miRNAs play important roles in diabetic pathology by regulating the expression of different genes. However, few studies focused on the roles of cyclin‐D2‐regulated miRNAs in endothelial dysfunction in T2DM.

The above studies indicated that high blood glucose can damage blood vessel function, and that cyclin D2 and miRNAs are involved in the pathology of vascular complications. In the present study, we treated rat aortic endothelial cells (RAOECs) with high glucose concentration to investigate the roles of cyclin‐D2‐regulated miRNAs in the vasculopathy. We found that cyclin D2 was up‐regualted and miR‐98 was downexpressed significantly in high glucose‐induced endothelial cells. We also revealed that miR‐98 negatively regulated the cyclin D2 levels, which are irregularly expressed in the aortas of T2DM rats.

## Materials and methods

### Cell culture and miRNA transfection

RAOECs (Shanghai Institute of Cell Biology, Shanghai, China) were cultured in DMEM (HyClone, Logan, UT, USA) supplemented with 10% foetal calf serum (HyClone, Logan, UT, USA) and 10 U/ml of penicillin–streptomycin (Sigma‐Aldrich, St. Louis, MO, USA). These cells were grown at 37°C with 5% CO_2_.

All transfections were performed in triplicate. For transfection, 1 × 10^6^ cells were transfected with 0.5 μg of miRNAs and 0.5 μg of pcDNA‐green fluorescent protein (GFP)‐untranslated region (UTR) plasmids in 2.5 μl of Lipofectamine 2000 (Invitrogen, Carlsbad, CA, USA) in accordance with the manufacturer's instructions. miR‐98 (mature miRNA) was used to suppress the expression of cyclin D2 in this study. The sense chain of miR‐98 was 5′‐UGAGGUAGUAAGUUGUAUUGUU‐3′. The sequence of antisense RNA (ASO‐98) used to inhibit the role of miR‐98 was 5′‐AACAAUACAACUUACUACCUCAC‐3′. Samples were analysed after 72 hrs.

### MTT assay

A modified colorimetric 3‐(4,5‐dimethylthiazol‐2‐yl)‐2,5‐diphenyltetrazolium bromide (MTT) assay was performed to study the role of miR‐98 in regulating RAOEC cell proliferation by using previously described methods 23, 24.

### Cell cycle detection

RAOECs (2×10^6^) were collected, resuspended and fixed by adding 4 ml of −20°C absolute ethanol. Cells were then centrifuged and resuspended in 1 ml of PBS. Up to 100 μl of DNase‐free, RNase A (200 μg/ml) was added and incubated at 37°C for 30 min. Approximately 1 ml of propidium iodide (PI) staining solution (50 μg/ml) was added to the cell pellet and mixed well. Cell cycle was detected *via* flow cytometry (Beckman Coulter, Inc., Brea, CA, USA).

### Western blot

At 24 hrs after treatment, RAOECs were lysed using the cell lysis buffer (Western of Beyotime, Shanghai, China) in accordance with the manufacturer's instruction. Protein concentration was determined using a BCA (Bicinchoninic Acid) Protein Assay kit (Beyotime). Total protein (30 μg) was loaded into individual lanes and separated in 10% SDS‐PAGE. Proteins were then transferred into polyvinylidene difluoride membranes (Bio‐Rad, Hercules, CA, USA). These membranes were blocked in Tris‐buffered saline containing 0.05% Tween‐20 (TBST) with 5% bovine serum albumin (BSA) for 1 hr, and then incubated overnight with mouse anti‐rat cyclin D2 antibody (1:400; Abcam, Austin, TX, USA) or rabbit anti‐rat RB/p‐RB/Bcl‐2/BAX/Caspase 9 antibody (1:400; Bioworld Technology, Inc., Minneapolis, MN, USA) in TBST at 4°C. Finally, the membranes were washed and incubated with horseradish peroxidase‐labelled goat antimouse or rabbit IgG (1:6000; Beijing Zhong Shan‐Golden Bridge Technology Co., Ltd., Beijing, China) and visualized by chemiluminescence (BeyoECLPlus; Beyotime). GAPDH or β‐Actin for was used as a control for each sample.

### Quantitative real‐time PCR

miRNAs were extracted from vascular tissues by using the mirVana^™^ miRNA isolation kit (Ambion, Carlsbad, CA, USA) in accordance with the manufacturer's instructions. The miRNAs were added with poly (A) tails by using poly (A) polymerase (Ambion). The cDNAs were synthesized as previously described 25. Real‐time quantitative polymerase chain reaction (qPCR) was performed with the following miR‐98 primers: forward, 5′‐TGAGGTAGTAAGTTGTAT‐3′ and reverse, 5′‐AACATGTACAGTCCATGGATG‐3′. Each qPCR reaction mix contained 0.5 μl of cDNA, 7.5 μl of sterile water, 1 μl of forward primer, 1 μl of reverse primer and 10 μl of SYBR Premix Ex Taq^™^ (Takara Biotechnology Co., Ltd., Dalian, China). The qPCR reaction was performed with the RG3000 system (Corbett Life Science, Mortlake, NSW, Australia) with the following thermal profiles: initial denaturation at 95°C for 3 min., followed by 38 cycles of denaturation at 95°C for 20 sec., annealing at 60°C for 20 sec., and extension at 72°C for 30 sec. The reference control was 5s rRNA. All experiments were repeated in triplicate.

### Immunofluorescence

Expression levels of cyclin D2 and p‐RB1 were determined by immunofluorescence staining. Cells grown on the slides were fixed in 1.5% paraformaldehyde. Cells on slides were permeabilized in 0.2% Triton X‐100, washed with PBS, and blocked in 5% BSA. Primary antibodies of mouse anti‐rat cyclin D2 (1:200; Santa Cruz Biotechnology, Inc., Santa Cruz, CA, USA) or rabbit anti‐rat p‐RB1 antibody (1:200; Bioworld Technology, Inc., Minneapolis, MN, USA) were added to the slides, which were incubated overnight at 4°C. After washing with PBS, slides were incubated with Alexa Fluor 594 Goat Anti‐Mouse IgG (H+L) (1:400 dilution; Life Technologies‐ Invitrogen) or Alexa Fluor 488 Goat Anti‐Rabbit IgG (H+L) (1:400 dilution; Life Technologies‐ Invitrogen) for 1 hr. Fluorescent images were captured under a microscope (DM6000B; Leica, Dresden, Germany).

### Animals and ethics

All animal experiments strictly followed the animal protocols and procedures established in Binzhou Medical University. A total of 25 male Sprague–Dawley rats, 6 weeks old and weighing 180–200 g, were obtained from the animal centre of Binzhou Medical University. All rats were kept in 20 cm × 40 cm × 60 cm cages, and maintained on a 12 hrs:12 hrs light–dark cycle. Before streptozotocin (STZ) treatment, 15 rats fed high fat diet and water initially for 4–5 weeks. Then these rats were treated with STZ (35 mg/kg) to induce diabetic rat model as previously reported 26. Ten age‐matched control rats were injected with the buffer alone, and fed standard rat diet and water throughout the experiment. All rats were killed; and the large artery between aorta ascendens and aorta thoracalis was immediately cut. All the tissues were frozen at −80°C and prepared for the miRNA extraction qPCR detection, and Western blot analysis.

### Construction of UTR report plasmid

Green fluorescent protein was digested from pEGFP‐N1 (TaKaRa, Otsu, Shiga, Japan) and cloned into pcDNA3.1(−) (Invitrogen) to form a pcDNA‐GFP vector as previously described 23. The 3′‐UTR (1603 bp) of cyclin D2 was amplified by PCR in an Eppendorf cycler by the following thermal profiles: 28 cycles of denaturation at 95°C for 45 sec., annealing at 60°C for 45 sec., and elongation at 72°C for 60 sec. The primers used were 5′‐AAGAGAGAGGCGTGTTCGTC‐3′ (forward); and 5′‐ATGGTTCAGTCGTGTGGTTG‐3′ (reverse). Afterward, 3′‐UTR was cloned into the T vector (TaKaRa) to construct a T‐UTR vector. The pcDNA‐GFP‐UTR plasmid was obtained by cutting the 3′‐UTR of cyclin D2 from the T‐UTR vector by *Eco*RI/*Sal*I and cloning GFP segment downstream of the pcDNA‐GFP plasmid by *Eco*RI/*Xho*I.

### GFP analysis

After transfection for 48 hrs, the GFP expression in RAOECs was first observed under a fluorescent microscope, and the percentage of GFP‐positive cells was estimated *via* flow cytometry (fluorescence‐activated cell sorting; Beckman Coulter, Inc.).

### Apoptosis detection

Cells were transfected with miR‐98 and miR‐98 inhibitors (ASO‐98) for 48 hrs. Cells were fixed in 70% ethanol at 4°C overnight, and then centrifuged at 1000 *g* for 10 min. Subsequently, cells were resuspended in residual ethanol by gently vortexing, and 0.5 ml PI staining solution (50 lg/ml) was added to each sample. Finally, the mixture was incubated for 30 min. and analysed *via* a flow cytometry (Beckman Coulter, Inc.).

### Statistical analysis

SPSS Statistics Client 22 (IBM) software was used to analyse the statistical significance of all results. anova was applied to compare different groups with respect to continuous variables. Group means were compared using an unpaired, two‐sided, Student's *t*‐test. A *P*‐value <0.05 was considered significant.

## Results

### High glucose concentration affects endothelial cell growth

**Figure 1:**
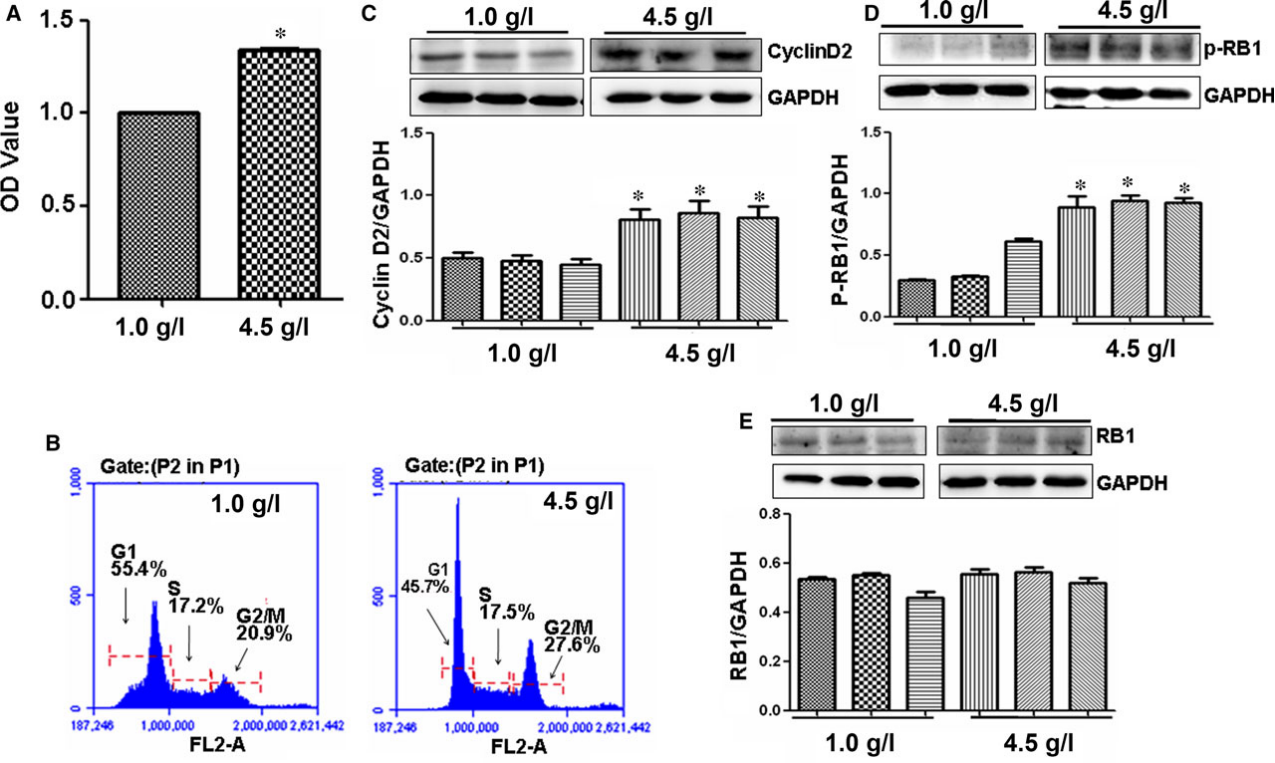
High glucose concentration affects vascular cell proliferation. (**A**) Cell proliferation. 4.5 g/l glucose significantly promoted RAOEC proliferation compared with 1.0 g/l glucose treatment. *P < 0.01 *versus* 1.0 g/l glucose treatment, *n* = 3 replicates. (**B**) Cell cycle. High level of glucose 4.5 g/l increased number of RAOECs in G2/M phase compared with 1.0 g/l glucose treatment. (**C**) Cyclin D2 expression. Western blot showed that cyclin D2 was overexpressed in 4.5 g/l glucose‐treated RAOECs compared with control treatment. *P < 0.01 *versus* 1.0 g/l glucose treatment, *n* = 3 replicates. (**D** and **E**) p‐RB and RB expressions. Western blot showed that p‐RB was increased in 4.5 g/l glucose‐treated RAOECs compared with control treatment, but no differences in RB levels were found between high and low concentrations of glucose‐treated cells. *P < 0.01 *versus* 1.0 g/l glucose treatment, *n* = 3 replicates.

High glucose levels in the blood can damage blood vessel function in T2DM, probably because of increased glucose intake by endothelial cells 3, 4. In the current study, RAOECs were treated with high (4.5 g/l) and low (1.0 g/l) concentrations of glucose *in vitro* to investigate the effects of increased glucose on endothelial cells of large artery, MTT assay results showed that 4.5 g/l glucose treatment significantly promoted RAOEC cell proliferation compared with 1.0 g/l glucose treatment (Fig. 1A). Cell cycle analysis further demonstrated that higher levels of glucose treatment induced more cells in G2/M phase (27.6%) than 20.9% in 1.0 g/l glucose‐treated cultures (Fig. 1B), which may have caused the difference in cell proliferation.

### High glucose concentration increases cyclin D2 and p‐RB1 expression

**Figure 2:**
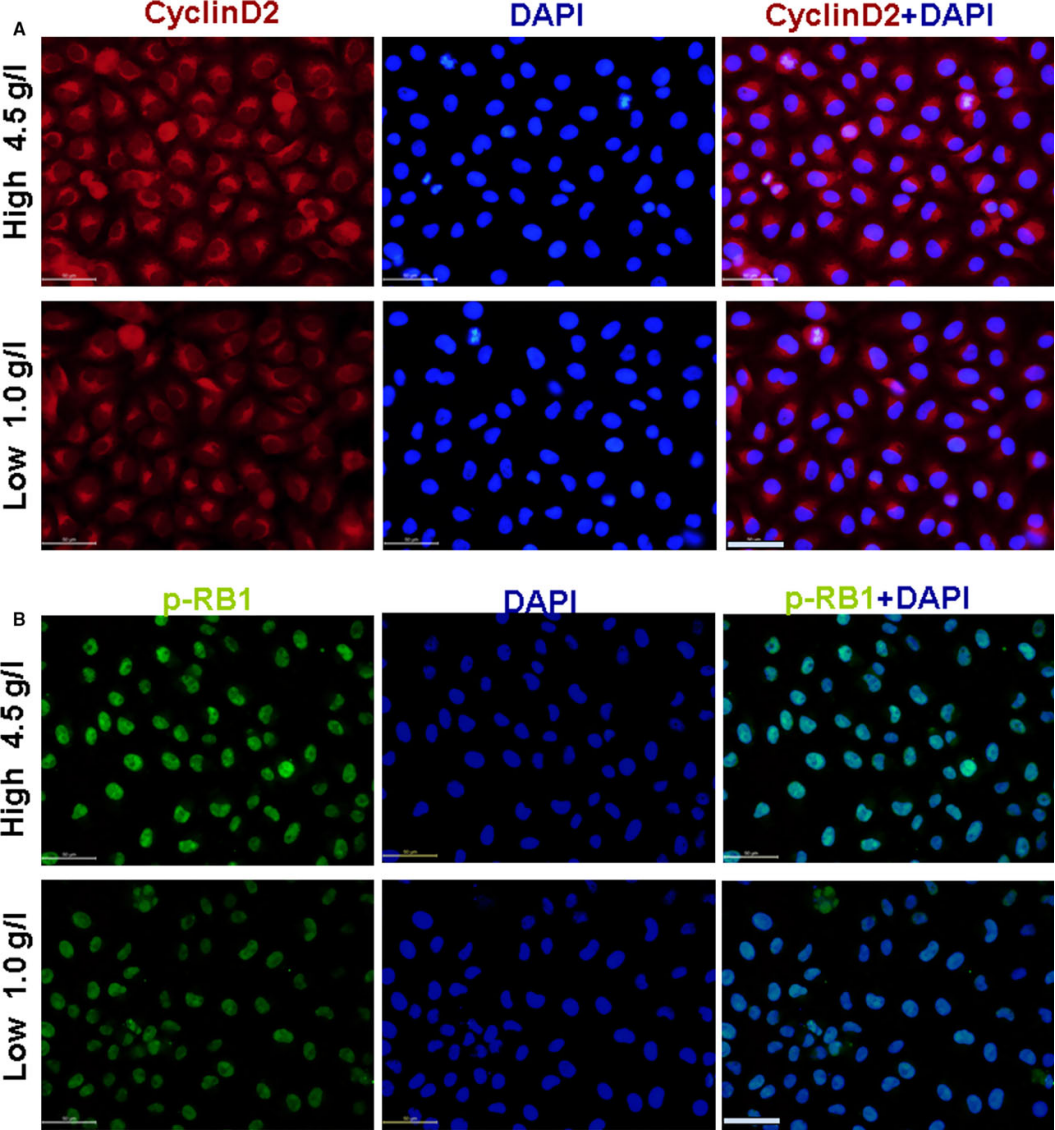
Effect of glucose on cyclin D2 and p‐RB1 expression. (**A**) Cyclin D2 expression (red). (**B**) p‐RB1 expression (green). Cyclin D2 and p‐RB1 expression increased in 4.5 g/l glucose‐treated RAOECs compared with control treatment, scale bar = 50 μm.

Cyclins, including cyclin D2, act as oscillators to drive the forces of cell cycle progression 27, in which cyclin D2‐induced phosphorylation of RB1 was vital 28. To study the mechanism of the high glucose concentration promoting endothelial cell proliferation, cyclin D2 and p‐RB1 levels in RAOECs treated with high glucose concentration were measured. Western blot results showed that high glucose levels significantly increased the expression of cyclin D2 and p‐RB1, but slightly affected the non‐phosphorated‐RB1 expression compared with low glucose concentration (Figs 1C–E and 2A and B).

### The expression of cyclin D2 and p‐Rb is regulated by miR‐98

**Figure 3:**
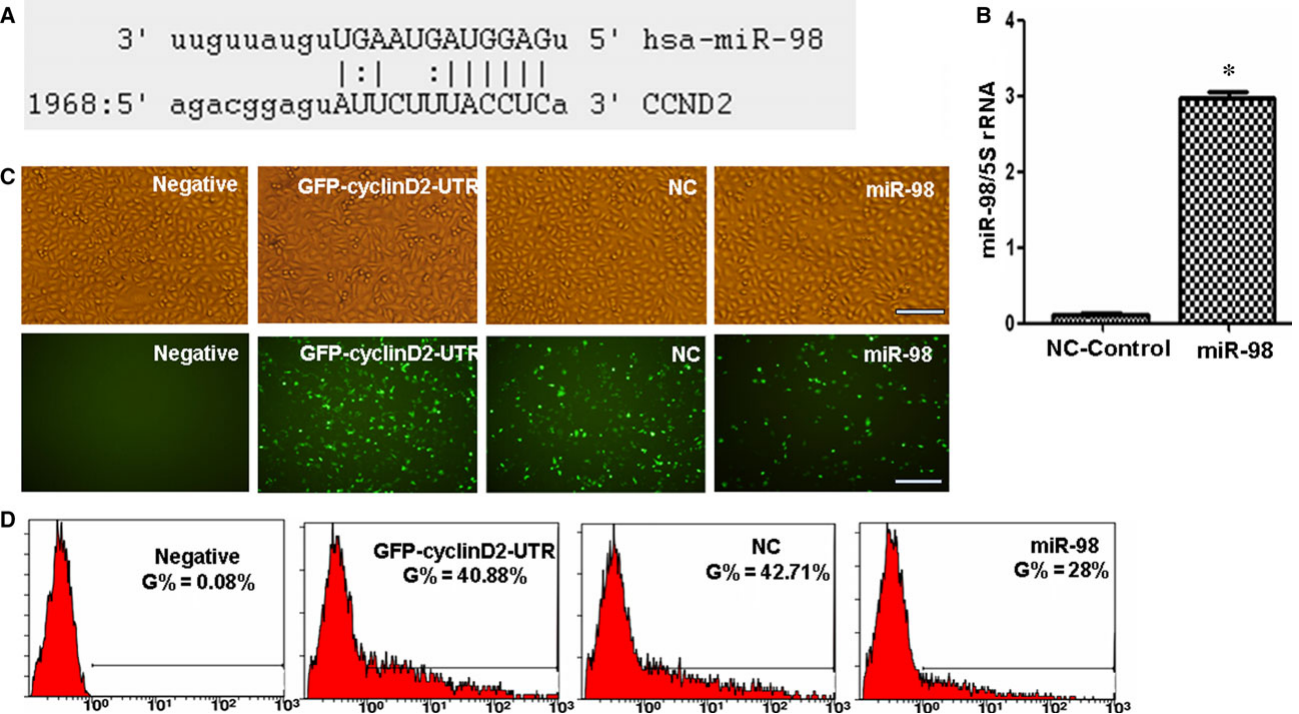
Cyclin D2‐3′UTR is targeted by miR‐98. (**A**) Sequences of cyclin D2 3′‐UTR targeted by miR‐98. (**B**) qPCR results showing that miR‐98 levels were higher in miR‐98‐treated RAOECs compared with NC‐control oligo treatment. *P < 0.01 *versus* control oligo treatment, *n* = 3 replicates. (**C**) Fluorescent microscopy image analysis results. Upper panel, phase‐contrast view under visible light; lower panel, fluorescence to reveal expression of GFP‐positive cells, scale bar = 100 μm. Fewer GFP‐positive endothelial cells were captured in miR‐98‐treated cultures compared with control oligo treatment under a microscope. (**D**) Flow cytometry analysis results revealed that the rate of GFP‐positive cells was significantly reduced in miR‐98‐transfected endothelial cells compared with NC‐control oligo‐treated cultures, *n* = 3 replicates.

Emerging evidence suggests that miRNAs are involved into the development of diabetes and its vascular complications 17, 18, 19. An online miRNA analysis software (http://www.microrna.org/microrna/getMirnaForm.do) showed that cyclin D2‐3′UTR is targeted by miR‐98 (Fig. 3A), as well as other miRNAs (data not shown). Then pcDNA‐GFP‐UTR reporter plasmid was constructed and transfected RAOECs with miR‐98 to verify whether cyclin D2‐3′UTR is regulated by miR‐98. The level of miR‐98 significantly increased in RAOECs after miR‐98 treatment (Fig. 3B). GFP expression significantly decreased in miR‐98‐treated RAOECs compared with that of in control oligos (NC)‐treated cultures (Fig. 3C). Fluorescence‐activated cell sorting results further revealed fewer GFP‐positive cells in miR‐98‐transfected cultures than in NC‐treated cultures (Fig. 3D), indicating that miR‐98 can regulate cyclin D2‐3′UTR activity.

**Figure 4:**
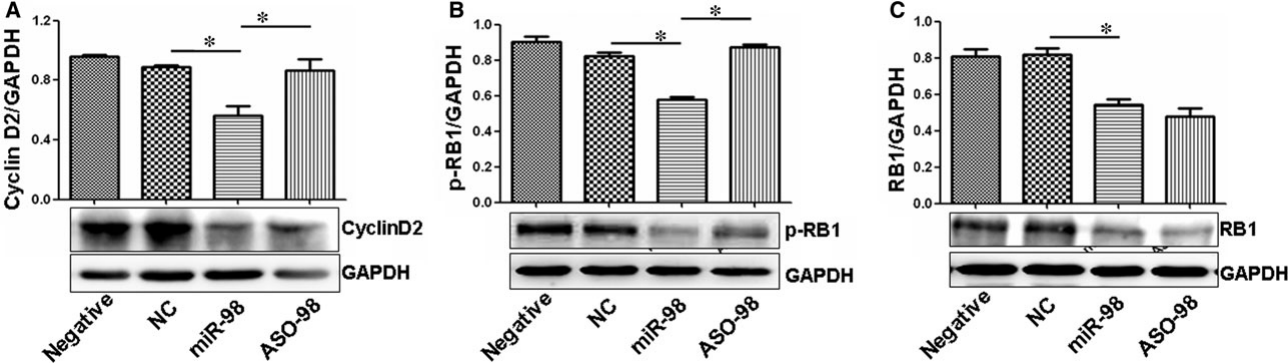
miR‐98 regulates cyclin D2 and RB expression. (**A**) Western blot results showed that miR‐98 treatment reduced cyclin D2 expression in endothelial cells compared with NC‐control treatment. Antisense RNA treatment inhibited the role of miR‐98 in suppressing cyclin D2 levels. *P < 0.05, miR‐98 treatment *versus* NC‐control or ASO‐98 treatment, *n* = 3 replicates. (**B**) miR‐98 treatment reduced p‐RB levels in endothelial cells compared with NC‐control treatment. Antisense RNA treatment increased p‐RB levels through miR‐98‐induced inhibition. *P < 0.05, miR‐98 treatment *versus* NC‐control or ASO‐98 treatment, *n* = 3 replicates. (**C**) RB expression was also reduced in miR‐98‐treated cells compared with NC‐control oligo treatment. *P < 0.05, miR‐98 treatment *versus* NC‐control treatment, *n* = 3 replicates.

To study the regulation of cyclin D2 by miR‐98, Western blot results confirmed that cyclin D2 expression decreased in miR‐98‐treated RAOECs compared with control oligo treatment (Fig. 4A). Levels of p‐RB1 and RB1 were also inhibited after miR‐98 treatment compared with control oligo treatment (Fig. 4B and C).

**Figure 5:**
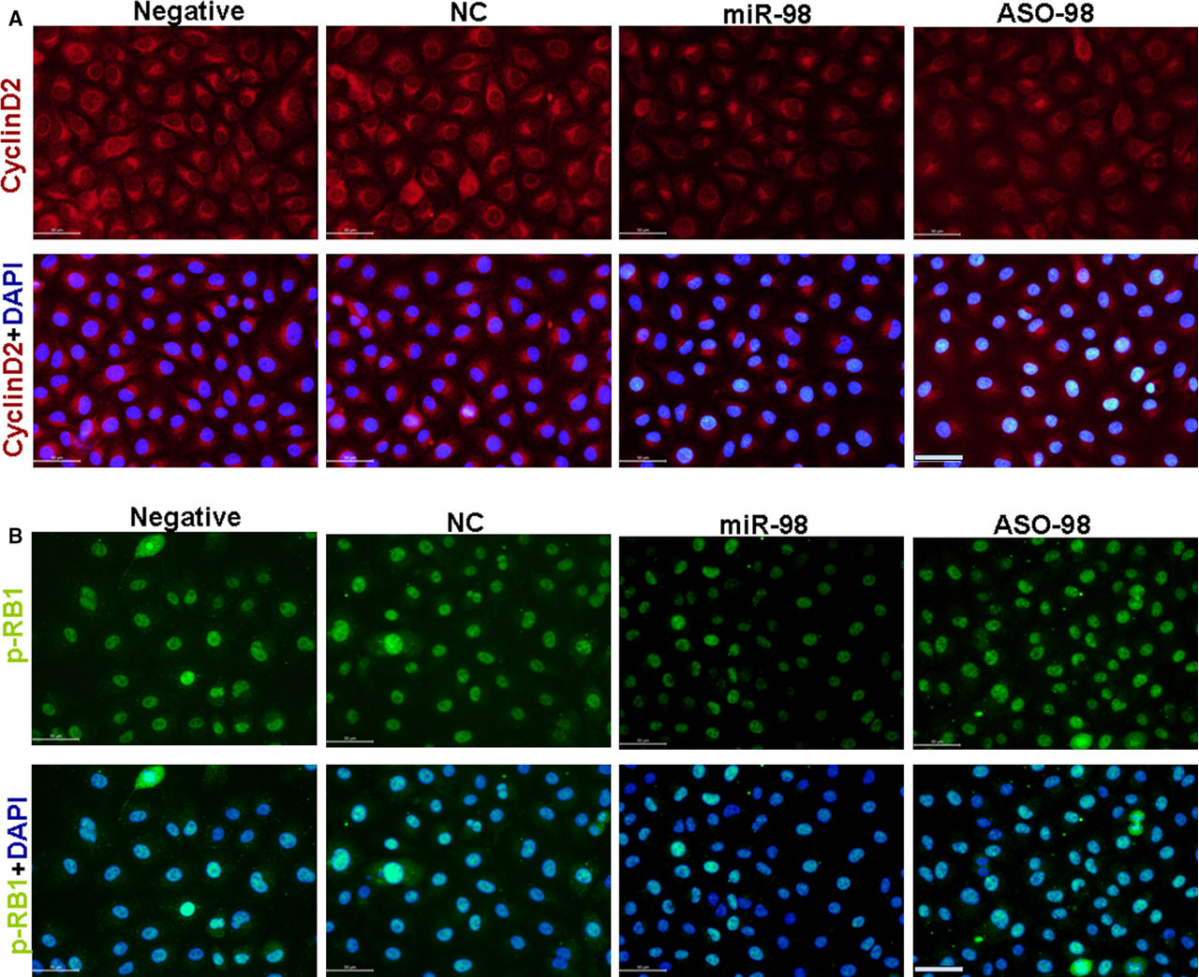
miR‐98 reduced cyclin D2 and p‐RB1 expression. (**A**) Cyclin D2 expression (red). (**B**) p‐RB1 expression (green). Expression of cyclin D2 and p‐RB1 decreased in miR‐98‐treated RAOECs compared with NC‐control treatment, which was recovered after ASO‐98 inhibited miR‐98 expression, scale bar = 50 μm, *n* = 3 replicates.

We also designed antisense oligos (Aso) inhibiting the expression of miR‐98 to confirm the regulating role of miR‐98 in cyclin D2 expression. When ASO‐98 inhibited the miR‐98 levels, the expression of cyclin D2 and p‐RB1 significantly increased compared with that in miR‐98‐treated RAOECs (Figs 4A, B and 5A, B), demonstrating that cyclin D2 was regulated by miR‐98.

### miR‐98 inhibits the proliferation of RAOECs

**Figure 6:**
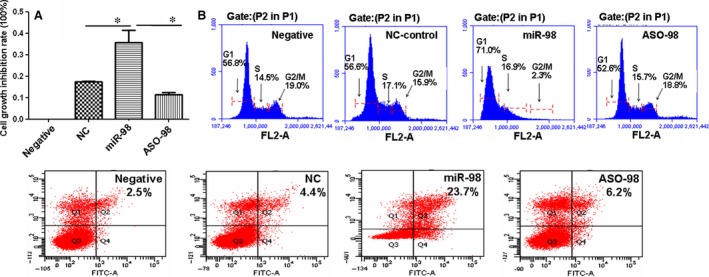
miR‐98 inhibits RAOEC growth. (**A**) MTT analysis results showing that the inhibition rate of cell proliferation was much higher in miR‐98‐treated endothelial cells compared with NC‐oligo‐treated cultures, and antisense RNA (ASO‐98) reduced the role of miR‐98 in suppression of cell growth. *P < 0.01, miR‐98 treatment *versus* NC‐control treatment or ASO‐98 treatment, *n* = 3 replicates. (**B**) Flow cytometry results showing that miR‐98 treatment induced G1 phase inhibition compared with control treatment. (**C**) Cell apoptosis. The apoptotic cell rate was 23.7% in miR‐98‐treated cells, which was much higher than that in NC‐ (4.4%) or ASO‐98‐treated cells (6.2%), *n* = 3 replicates.

The above results demonstrated that miR‐98 regulated cyclin D2 expression, which is up‐regulated in proliferating cells 13. Thus, we determined whether RAOEC cell proliferation was affected by miR‐98 through regulating cyclin D2 expression. First, we found that miR‐98 treatment significantly inhibit RAOEC proliferation (Fig. 6A). When miR‐98 inhibited cyclin D2 expression, 71% RAOECs were arrested in G1 phase in miR‐98‐treated cultures, compared with NC‐control treatment (56.6%, Fig. 6B). The RAOEC growth retardation induced by miR‐98 was ameliorated when ASO‐98 inhibited the miR‐98 levels (Fig. 6B). Moreover, we found that miR‐98 can induce RAOEC apoptosis, and more apoptotic RAOECs were found in miR‐98‐treated cultures than in NC‐control‐treated cells (Fig. 6C). Fewer apoptotic cells were measured in ASO‐98 treatment than in miR‐98 treatment (Fig. 6C).

### The process of miR‐98 inhibiting RAOEC proliferation involves apoptotic gene expression

**Figure 7:**
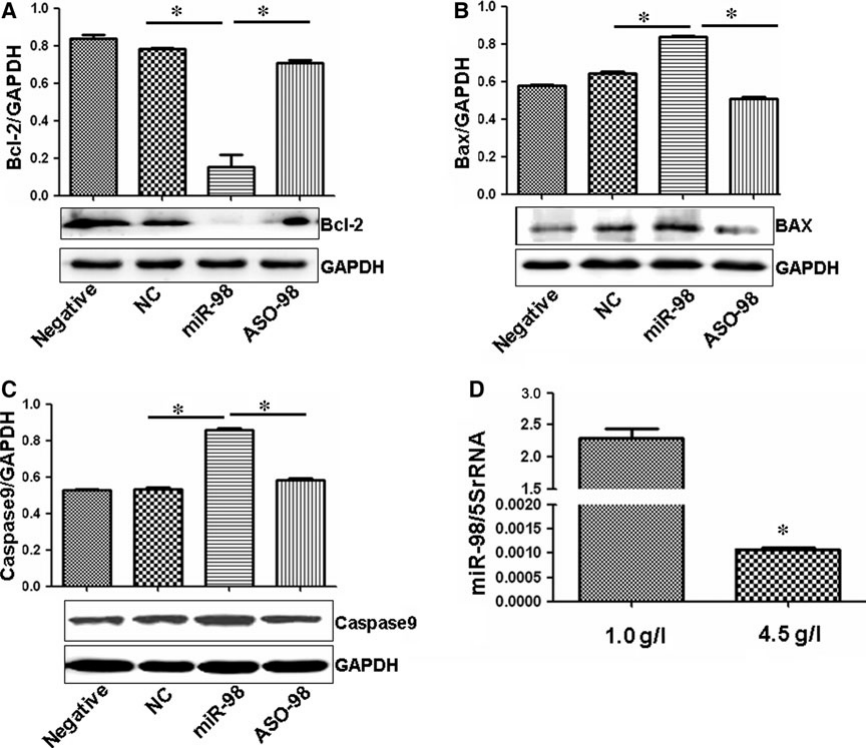
Several apoptotic genes expression affected by miR‐98. (**A**) Bcl‐2 expression was much lower in miR‐98‐treated endothelial cells compared with controls, and ASO‐98 increased the levels of Bcl‐2 compared with miR‐98 treatment. The ratio of Bcl‐2/GAPDH is shown at the top of the gel. *P < 0.01 *versus* NC‐control treatment or ASO‐98 treatment, *n* = 3 replicates. (**B**) Bax expression was much higher in miR‐98‐treated endothelial cells compared with controls, which was ameliorated by ASO‐98 treatment. The ratio of Bax/GAPDH is shown at the top of the gel. *P < 0.01 *versus* NC‐control treatment or ASO‐98 treatment, *n* = 3 replicates. (**C**) Caspase 9 expression was much higher in miR‐98‐treated endothelial cells compared with controls, which was ameliorated by ASO‐98 treatment. The ratio of Caspase 9/GAPDH is shown at the top of the gel. *P < 0.01 *versus* NC‐control treatment or ASO‐98 treatment, *n* = 3 replicates. (**D**) qPCR showing significantly lower miR‐98 levels in 4.5 g/l glucose‐treated RAOECs compared with 1.0 g/l glucose‐treated cultures. *P < 0.01 *versus* 1.0 g/l glucose treatment, *n* = 3 replicates.

We measured the expression of Bcl‐2, Bax and Caspase 9 expression to investigate the mechanism of miR‐98 suppression in RAOEC growth. We found that Bcl‐2 levels were lower in miR‐98‐transfected cells than in NC‐controls (Fig. 7A), whereas Bcl‐2 was overexpressed after ASO‐98 suppressed the miR‐98 levels. Bax and Caspase 9 levels were higher in miR‐98‐treated cells than in NC‐control‐treated cultures (Fig. 7B and C), and ASO‐98 treatment verified the role of miR‐98 in regulating Bax and Caspase 9 expression. These results showed that the role of miR‐98 in suppressing the growth of RAOEC might be related to regulation the expressions of Bcl‐2, Bax and Caspase 9 expression.

### High concentration glucose decreases miR‐98 levels in ROAECs

The above results high concentration of glucose promoted RAOEC proliferation *via* increasing cyclin D2 levels, and cyclin D2 expression was negatively regulated by miR‐98. We then studied whether high concentration of glucose affected miR‐98 expression in RAOECs. Interestingly, in contrary to the increased cyclin D2 expression, qPCR showed that the levels of miR‐98 decreased in 4.5 g/l glucose‐treated cells compared with low concentration of glucose treatment (Fig. 7D). These results indicated that cyclin D2 and miR‐98 might play important roles in the effects of increased glucose on endothelial cell growth.

### Cyclin D2 increase and miR‐98 decrease in aortas of STZ‐induced diabetic rats

**Figure 8:**
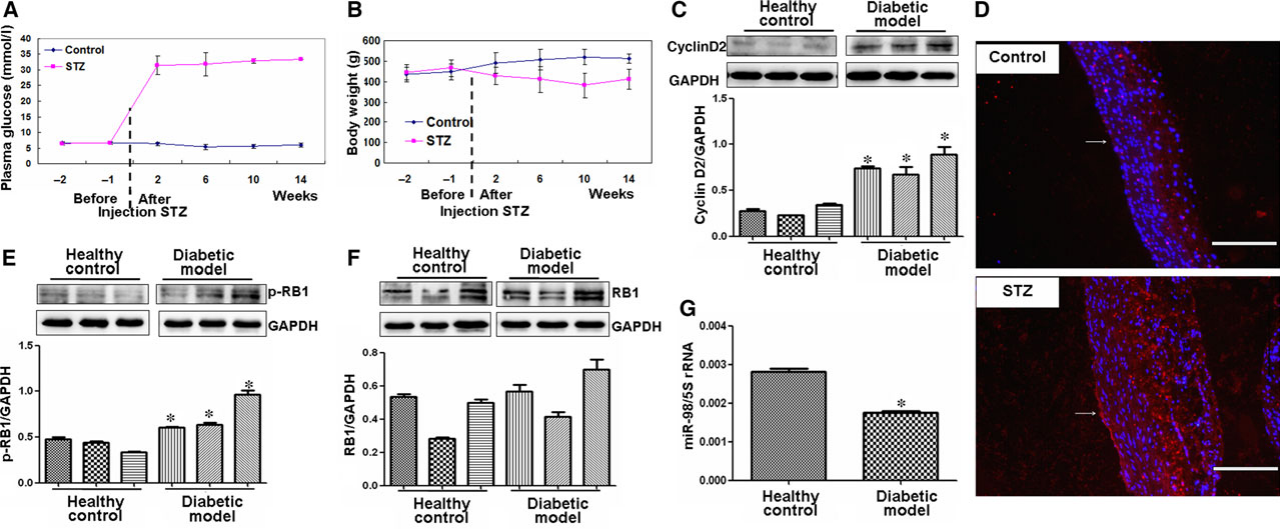
Expression of cyclin D2‐related factors in STZ‐induced diabetic rat model. (**A** and **B**) The changes of plasma glucose and bodyweights of STZ rats. Two weeks after STZ treatment, the plasma glucose significantly increased, but the bodyweight significantly reduced in STZ‐induced diabetic rats (*n* = 15) compared with control rats (*n* = 10). (**C**) Cyclin D2 expression. Cyclin D2 levels increased in aortas of diabetic rat model (*n* = 3) compared with those in healthy control rats (*n* = 3). *P < 0.05 *versus* control rats. (**D**) Cyclin D2 expression (red). Expression of cyclin D2 significantly increased in endothelial cells and smooth muscle cells of STZ‐induced diabetic rat aortas compared with control rats, scale bar = 200 μm. Arrow, indicating the expression of cyclin D2 in endothelial cells. (**E** and **F**) p‐RB and RB expressions. p‐RB levels significantly increased in aortas of diabetic rat model (*n* = 3) compared with those in control rats (*n* = 3). *P < 0.05 *versus* control rats. (**G**) miR‐98 expression decreased in aortas of diabetic rat model (*n* = 5) than that in healthy control rats (*n* = 5). *P < 0.05 *versus* control rats.

Streptozotocin‐induced animal model of diabetes demonstrates an important pathophysiological event indicative of endothelial cell dysfunction in aortas 26. To further investigate the roles of cyclin D2 and miR‐98 in pathophysiological event of diabetic rat aortas, we established STZ‐induced diabetic rat model as previously reported 26. The plasma glucose levels were all significantly higher in STZ‐induced diabetic rats (*n* = 15) compared with those in age‐matched control rats (*n* = 10), but the bodyweights were significantly lower in STZ rats (*n* = 15) compared with control rats (*n* = 10, Fig. 8A and B). Then we detected the expression of cyclin D2 and miR‐98 in aortas of diabetic rats. Similar to the expression of cyclin D2 and miR‐98 detected in the *in vitro* study, the levels of cyclin D2 significantly increased in endothelial cells and smooth muscle cells of STZ‐induced diabetic rat aortas compared with those in the healthy control rats (Fig. 8C and D). p‐RB1 were also obviously enhanced in endothelial cells and smooth muscle cells of STZ‐induced diabetic rat aortas compared with those in the healthy control rats (Fig. 8E and F). The expression of miR‐98 significantly decreased in STZ‐induced rat aortas compared with that in the healthy control rats (Fig. 8G), indicating that cyclin D2 and miR‐98 played important roles in pathophysiological event of diabetic rat aortas.

## Discussion

Proliferation of endothelial cells is an important feature in the pathogenesis of vascular complications 29, 30. Wang *et al*. showed that scutellarin treatment would inhibit high glucose‐induced proliferation in human retinal endothelial cells 30. Chen *et al*. found that 30 mM glucose significantly promoted the migration and proliferation of endothelial cells, which was blocked by 1 μg/ml adrenomedullin 29. Similarly, we demonstrated that 4.5 g/l (25 mM) glucose significantly promoted RAOEC proliferation compared with 1.0 g/l (~5 mM) glucose treatment, indicating that high concentration of glucose is involved in the dysfunction of endothelial cells.

High glucose concentration is important in the senescence of endothelial cells or endothelial dysfunction in T2DM rats 31 because it can regulate miRNA activity. For example, high glucose concentration reduced EZH2 binding to the miRNA (miR‐101) locus, whereas EZH2‐β overexpression inhibited miR‐101 promoter activity in human foetal endothelial cells of the umbilical cord vein 32. In the present study, we found that high concentration of glucose reduced the levels of miR‐98, which further affected cyclin D2 expression in RAOECs and induced RAOEC apoptosis. The apoptotic mechanism might be related to miR‐98 regulation of Bcl‐2, Bax and Caspase 9 expression. Our results demonstrated that cyclin D2 and miR‐98 played important roles in the increased glucose effects on endothelial cell growth.

Three different D‐type cyclins, namely, cyclins D1, D2 and D3, show significant amino acid similarities. Ablation of all three D‐type cyclins in embryonic mice leads to cardiovascular abnormalities and death 33. Among them, cyclin D2 is important in cell cycle progression from the G1 phase to S phase 34. Similarly, we demonstrated that cyclin D2 levels were enhanced in RAOECs induced with high glucose concentration, which promoted fewer cells in G1 phase and more cells in S phase. Our results indicated that cyclin D2 might be involved in the progression of cardiovascular abnormalities associated with diabetes.

Restriction point transit, the key regulatory step in the cell division, is largely regulated by the activity of CDK4 and its obligate cofactors, the D‐type cyclins 35, 36. Cyclin D2 protein forms a complex with CDK 4 or CDK 6, which then translocates to the nucleus, where the active cyclin D/CDK complex phosphorylates members of the retinoblastoma gene family to initiate a new round of cell cycle activity. This cascade of events leads to G1 to S phase transition 37. Under high glucose concentrations, we found that overexpressed cyclin D2 increased the level of p‐RB, which promoted G1 to S phase transition of ROAECs.

microRNAs participate in various cardiovascular cell processes, such as development, proliferation and apoptosis, which are related to numerous cardiovascular diseases, including coronary heart disease, myocardial infarction and hypertension 38, 39. In the process of vascular diseases, circulating platelets in the blood may directly adhere to vascular lesion sites and release various regulatory factors, including miRNAs, thereby accelerating disease progression 40. Some scientists 16, including our group, have suggested that cyclin D2 is involved in the pathology of vascular complications. Previous studies on different cells have shown several miRNAs, such as miR‐26a 41, miR‐302b, miR‐497 42 and miR‐198^43^, that can regulate cell proliferation by targeting cyclin D2. Interestingly, we predicted that cyclin D2‐3′UTR is targeted by miR‐98 and other miRNAs. We then demonstrated that a novel miRNA (miR‐98) can regulate cyclin D2 in ROAECs. When suppressing cyclin D2 levels by miR‐98, we demonstrated that RAOEC growth was blocked, which was shown by Pan's study 44, suggesting that propranolol decreased cyclin D2 expression, blocking norepinephrine‐induced endothelial cell cycle progression. We also revealed that miR‐98 can induce RAOEC apoptosis, the process of which might be related to the regulation of Bcl‐2, Bax and Caspase 9 expression.

Thus, we report that cyclin D2 increases and miR‐98 decreases in endothelial cells of diabetic rat large artery. Similarly, our *in vitro* study showed that high glucose concentration induces cyclin D2 overexpression and miR‐98 downexpression in the RAOECs. We also showed that miR‐98 inhibits human endothelial cell growth by regulating cyclin D2. Overall, our work provides essential information regarding novel therapeutic targets for vascular complications of T2DM.

## Conflicts of interest

The authors declare that they have no conflicts of interest.
